# Supplementary material for: Domain wall motion-driven magnetic convolutional accelerator
Source: Nat Commun. 2026 Jan 14;17:1281. doi: 10.1038/s41467-025-68041-4 (PMC12868888; doi:10.1038/s41467-025-68041-4)
Supplement: Supplementary file 1 — Supplementary Information [file 41467_2025_68041_MOESM1_ESM.pdf]

Supplementary Information

Domain Wall Motion-Driven Magnetic Convolutional Accelerator

Authors

Bingqian Dai[1,*,†], Tianyi Wang[1,*], Albert Lee[1,*], Shijie Xu[1,*], Chin-Chung Chen[1,2], Kin Wong[1], Dingyi Li[1], Malcolm Jackson[1], Yang Cheng[1], Puyang Huang[1], Yaochen Li[1], Chao Yun[3], Qingyuan Shu[1], Haoran He[1], Lixuan Tai[1], Hanshen Huang[1], Tien-Kan Chung[2], Yanglong Hou[3,†], and Kang L. Wang[1,†]

Affiliations

[1]*Department of Electrical and Computer Engineering, Physics and Astronomy, and Material Science and Engineering, University of California, Los Angeles, California 90095, United States*

[2]*Department of Mechanical Engineering, National Yang Ming Chiao Tung University, Hsinchu, Taiwan*

[3]*School of Materials, Shenzhen Campus of Sun Yat-sen University, Shenzhen 518107, China*

*These authors contributed equally to this work.

Corresponding author. E-mail: †bdai@g.ucla.edu, †hou@sysu.edu.cn, †wang@ee.ucla.edu

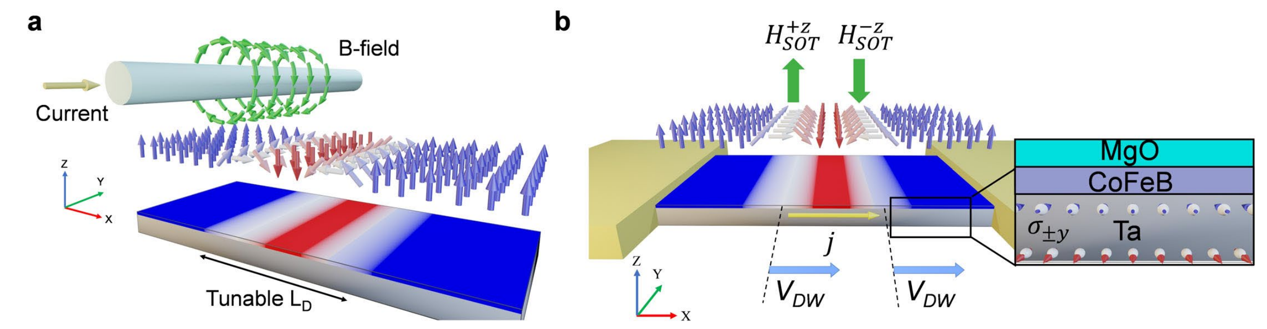

Supplementary Figure 1 | Physical principles of the MCA. (a) Domain and DW nucleation induced by a current-generated Oersted field. The domain length L_D can be tuned by adjusting the Oersted field. **(b)** DW motion driven by SOT. The effective fields generated by SOT are indicated by green arrows. The DW velocity (V_{DW}) is aligned with the direction of the applied current j. The right schematic shows the material stack used in the device. Spin orientations ($\sigma_{\pm y}$) are illustrated as blue or red arrows (spin direction) associated with white spheres (representing electrons).

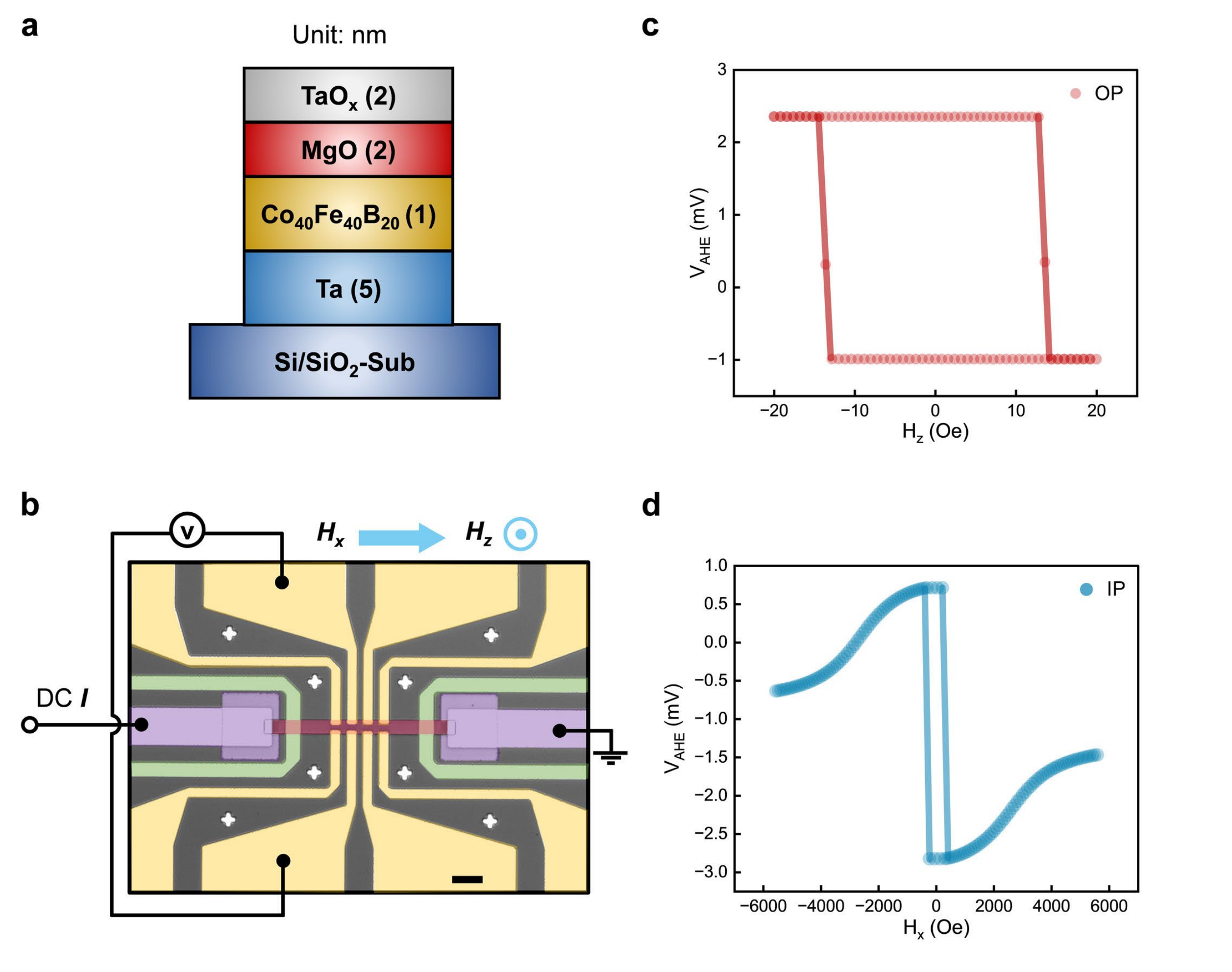

Supplementary Figure 2 | Material platform and magnetic characterization. (a) Schematic of the material stack used for the MCA platform. **(b)** Measurement setup for AHE characterization. The color coding follows that of Fig. 2c. A DC current I is applied along the sample, and the transverse Hall voltage V_{AHE} is measured while sweeping either the out-of-plane (OP) magnetic field H_z or the in-plane (IP) magnetic field H_x. **(c)** AHE loop measured under H_z sweeping. A characteristic square hysteresis loop is observed, consistent with perpendicular magnetic anisotropy (PMA) in the thin film. **(d)** AHE loop measured under H_x sweeping.

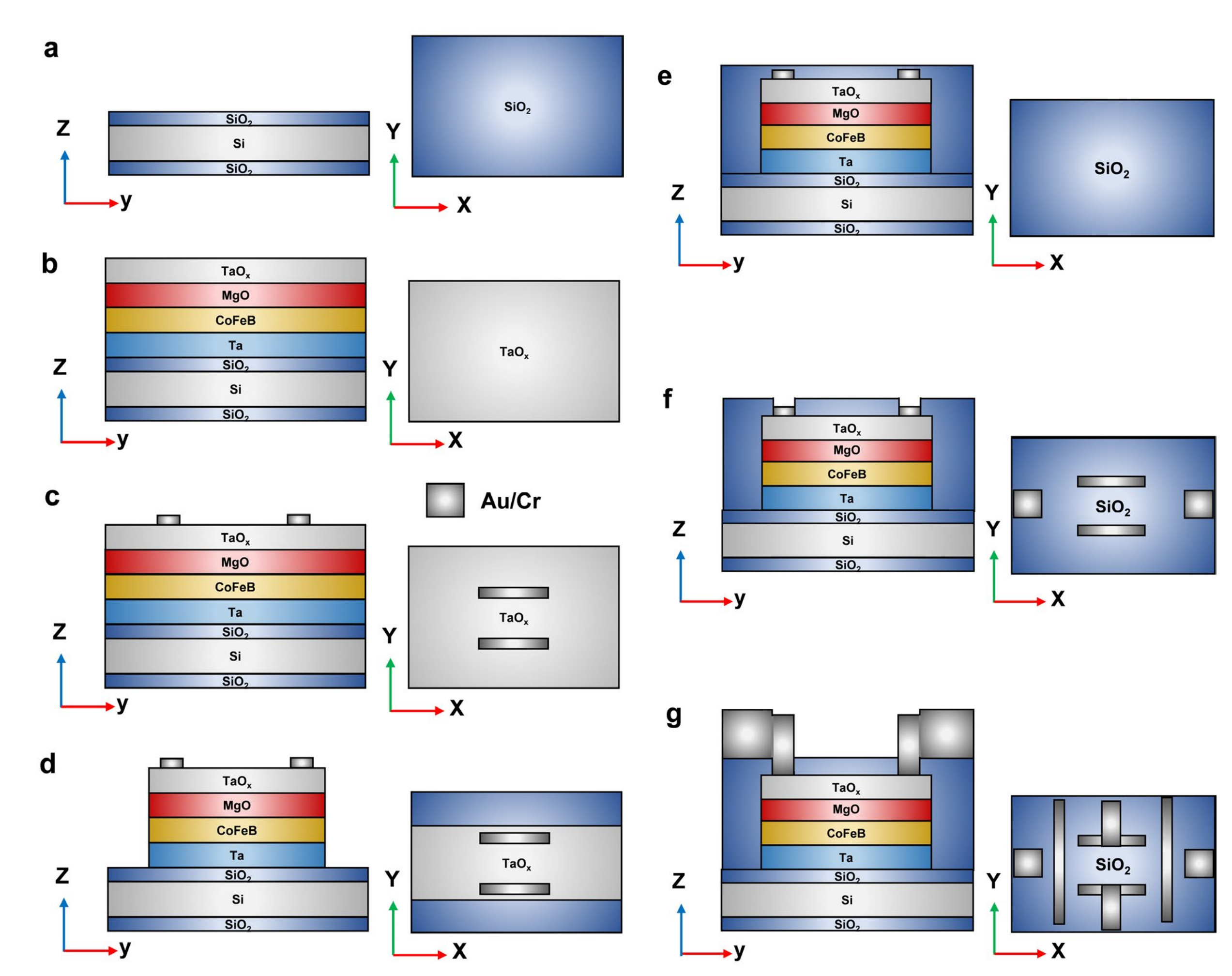

Supplementary Figure 3 | Device fabrication process. (a) Silicon (Si) substrate with thermally grown silicon dioxide (SiO_2) layer. **(b)** Deposition of the Ta/CoFeB/MgO/TaOx multilayer stack. **(c)** Photolithography and metal evaporation for forming Au/Cr electrodes. **(d)** Photolithography and dry etching to define the magnetic strip. **(e)** Deposition of SiO_2 insulating layer by evaporation. **(f)** Photolithography and wet etching to create Via openings. **(g)** Photolithography and metal evaporation for the Writing Line, Shifting Pads, and Hall Pads.

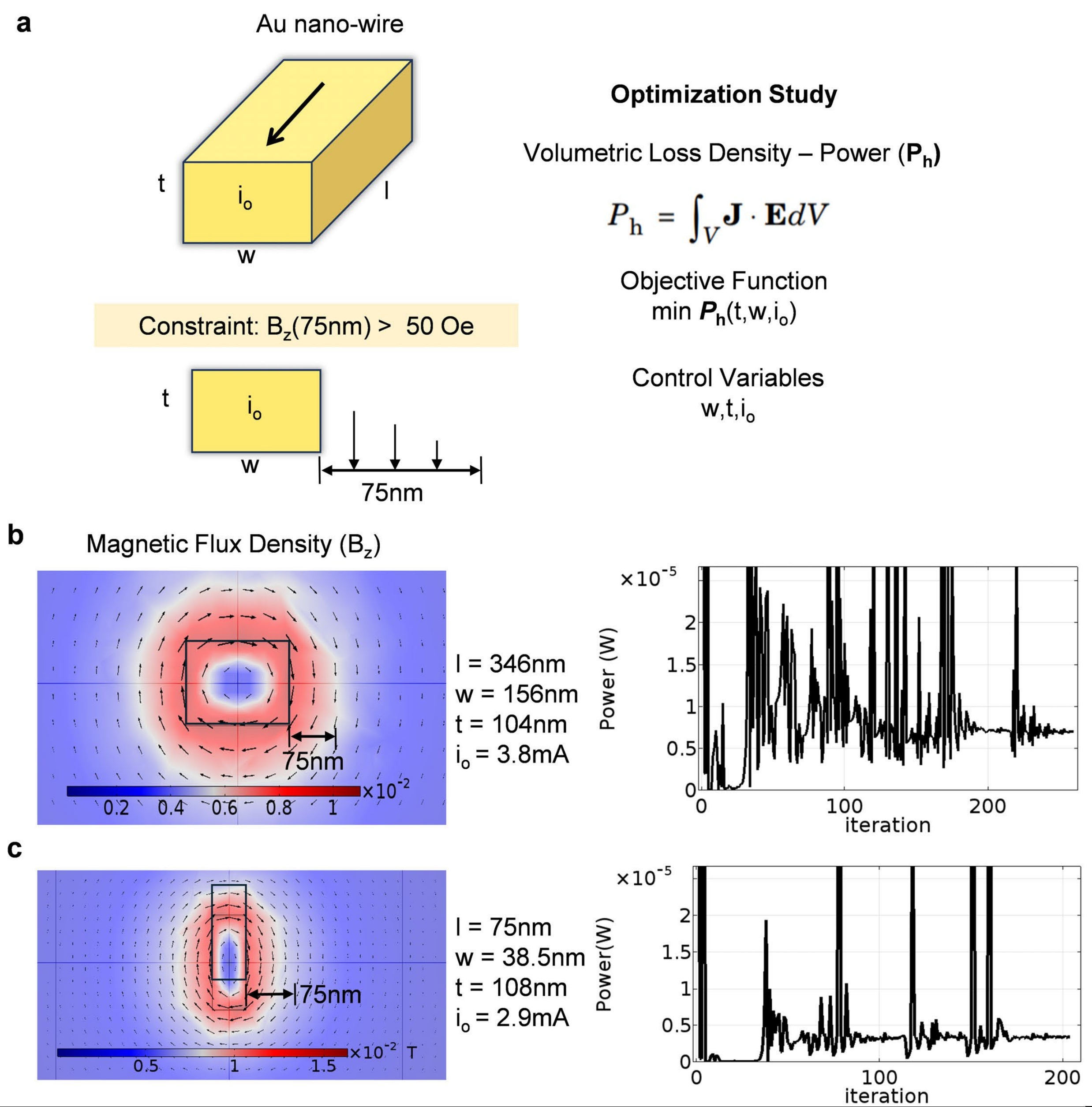

Supplementary Figure 4 | Spatial distribution of current-generated Oersted field and power optimization. (a) Geometry of the gold wire used for Oersted field generation and parameters for optimization study. **(b)** Results for wire length $l = 346\ nm$. Left: spatial distribution of the current-generated Oersted field based on optimized parameters. Right: power consumption as a function of optimization iteration. **(c)** Results for wire length $l = 75\ nm$.

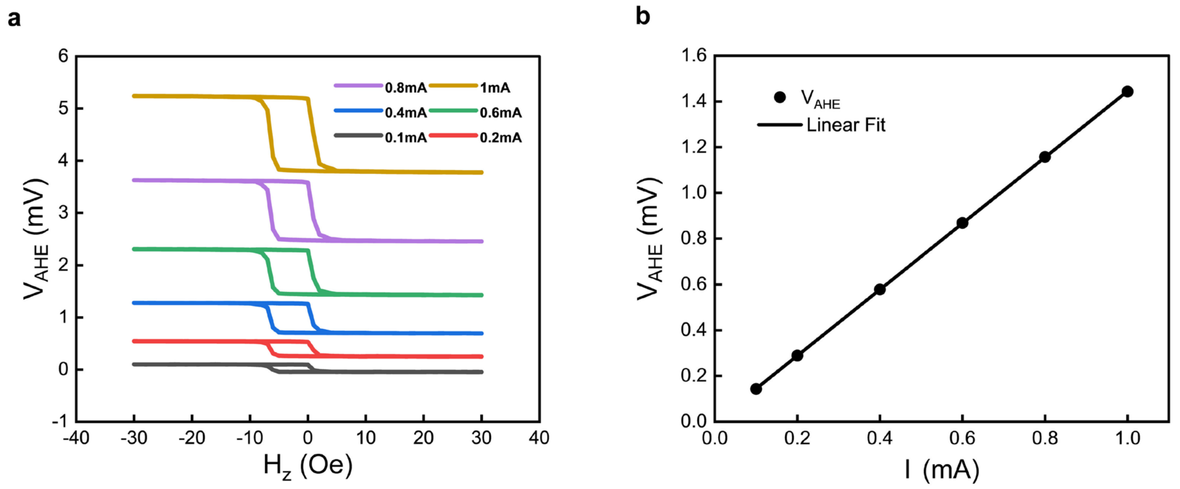

Supplementary Figure 5 | Current dependence of anomalous Hall voltage. (a) AHE hysteresis loops measured as a function of applied current I. All measurements are performed on the same device. **(b)** Plot of V_{AHE} versus I, showing a linear dependence.

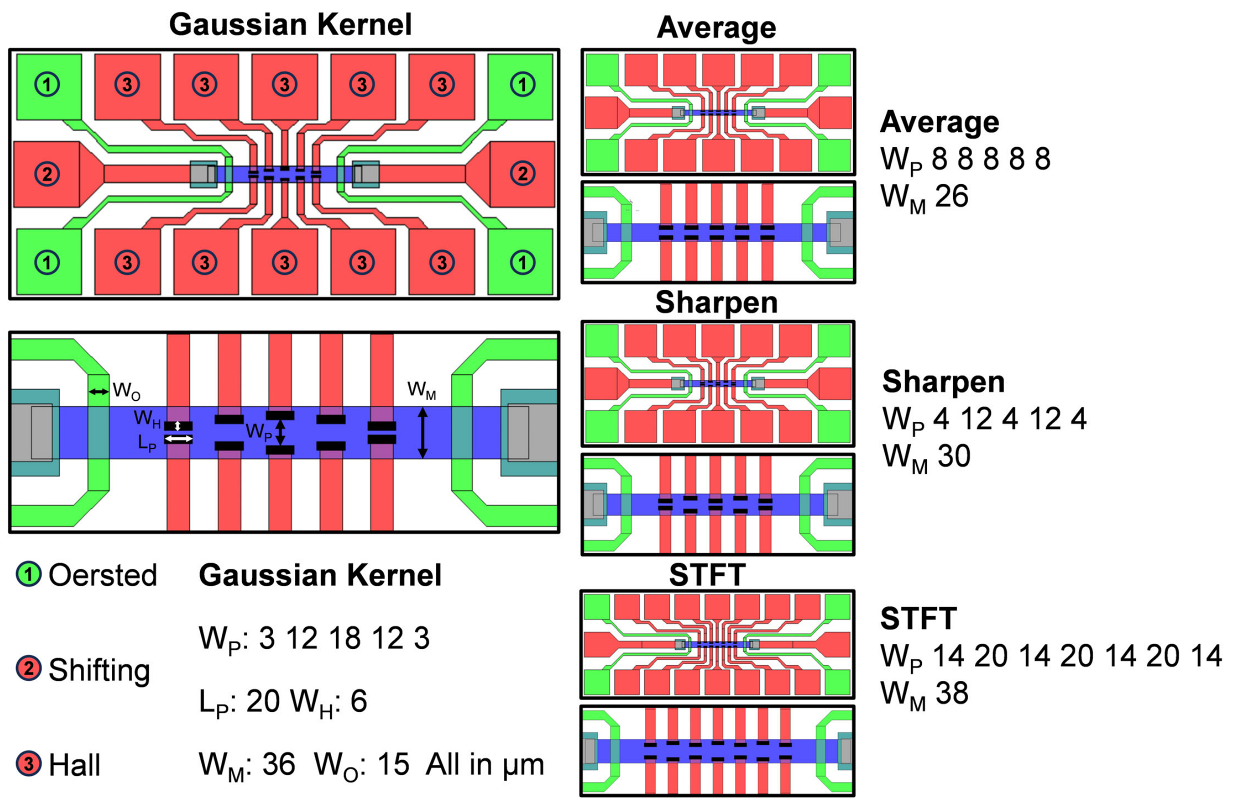

Supplementary Figure 6 | Dimensions and patterns of the MCA. Dimension specifications of the MCA devices designed for different kernel functionalities.

For the Gaussian kernel: distance between pairs of Hall pads $W_P = 3 - 12 - 18 - 12 - 3\ \mu m$; Hall pads length $L_P = 20\ \mu m$; Hall pads width $W_H = 6\ \mu m$; Magnetic strip width $W_M = 36\ \mu m$; Oersted field channel width $W_O = 15\ \mu m$.

For the Average kernel: $W_P = 8 - 8 - 8 - 8 - 8\ \mu m$; $L_P = 20\ \mu m$; $W_H = 6\ \mu m$; $W_M = 26\ \mu m$; $W_O = 15\ \mu m$.

For the Sharpen kernel: $W_P = 4 - 12 - 4 - 12 - 4\ \mu m$; $L_P = 20\ \mu m$; $W_H = 6\ \mu m$; $W_M = 30\ \mu m$; $W_O = 15\ \mu m$.

For the STFT kernel: $W_P = 14 - 20 - 14 - 20 - 14 - 20 - 14\ \mu m$; $L_P = 20\ \mu m$; $W_H = 6\ \mu m$; $W_M = 38\ \mu m$; $W_O = 15\ \mu m$.

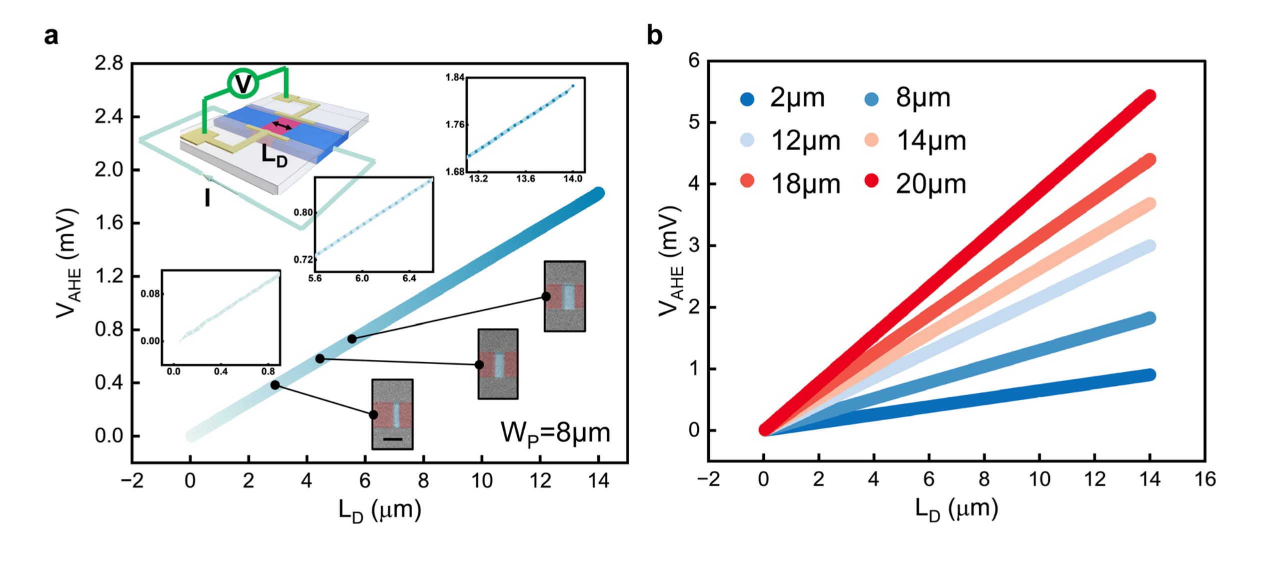

Supplementary Figure 7 | Linearization of domain length response. (a) Linearized plot of V_{AHE} versus L_D, obtained by 256-bin averaging of the raw data from Fig. 2g. The top insets show the experimental setup and three zoom-in views of the linearized curve, where the error bands become more visible. The bottom inset shows three snapshots during the domain length (L_D) expansion process. **(b)** Linearized plots of V_{AHE} versus L_D for different W_p values. A total of 382 W_p configurations were tested and linearized; six representative examples are shown here.

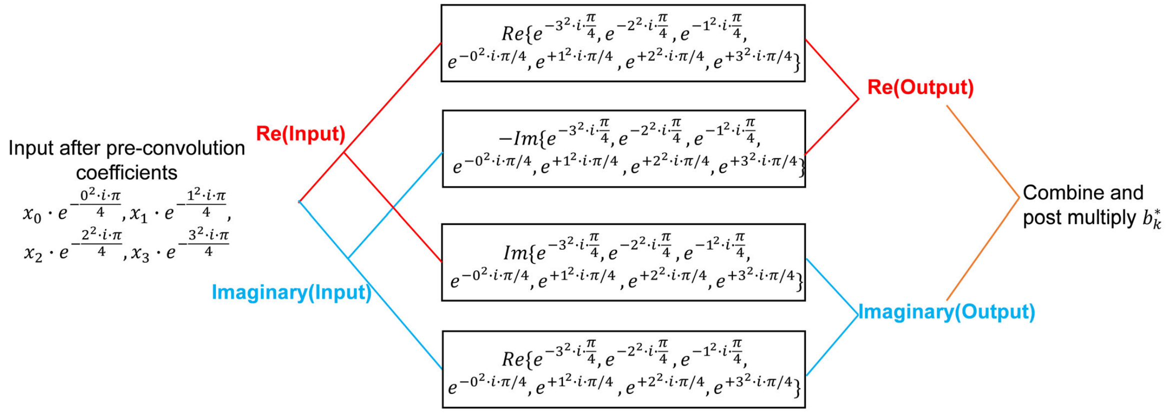

Supplementary Figure 8 | Mapping of DFT to the MCA device. Complex number processing is performed by decomposing the input signal into real and imaginary parts, each handled by separate MCA devices. Four MCA devices are used in total to process both components. The combined output is then post-multiplied by ${b_k}^*$ to yield the Fourier-transformed signal X_k.

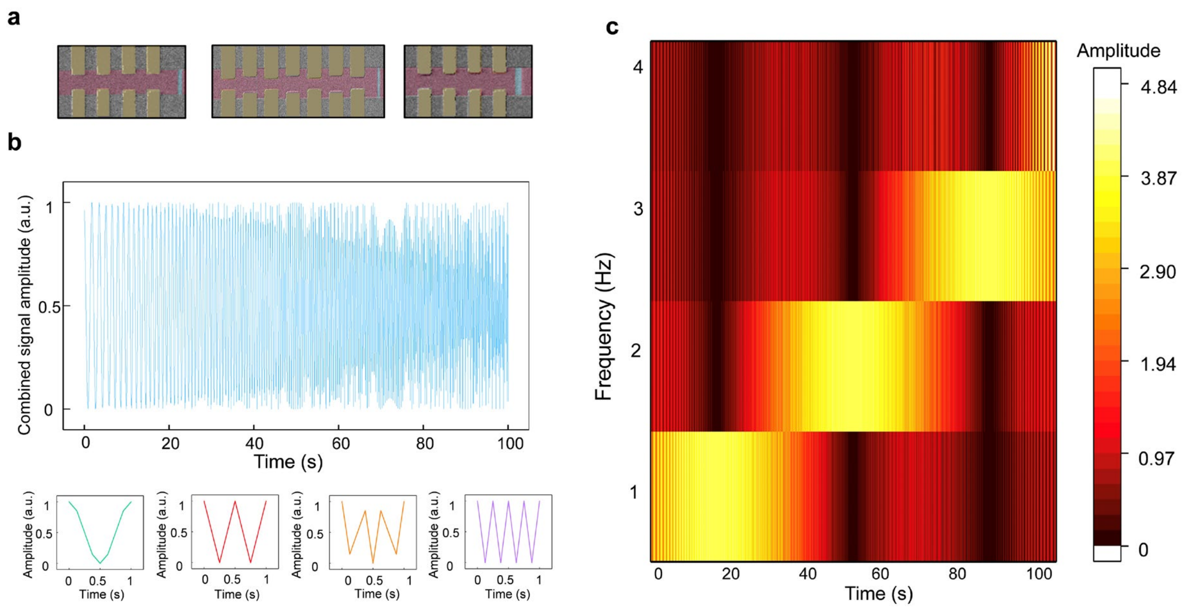

Supplementary Figure 9 | 8-point STFT implementation using MCA devices. (a) A 15-Hall-pad structure constructed from three MCA devices. **(b)** A time-varying input signal $f(t) = \frac{1}{2}\{\cos[2\pi(1 + 3 * 0.01 * t) \times t] + 1\}$ is fed into the device. The lower plots show four representative frequencies sampled at different time intervals. **(c)** Resulting STFT spectrum. The color map captures the amplitude evolution of $f(t)$ over time.

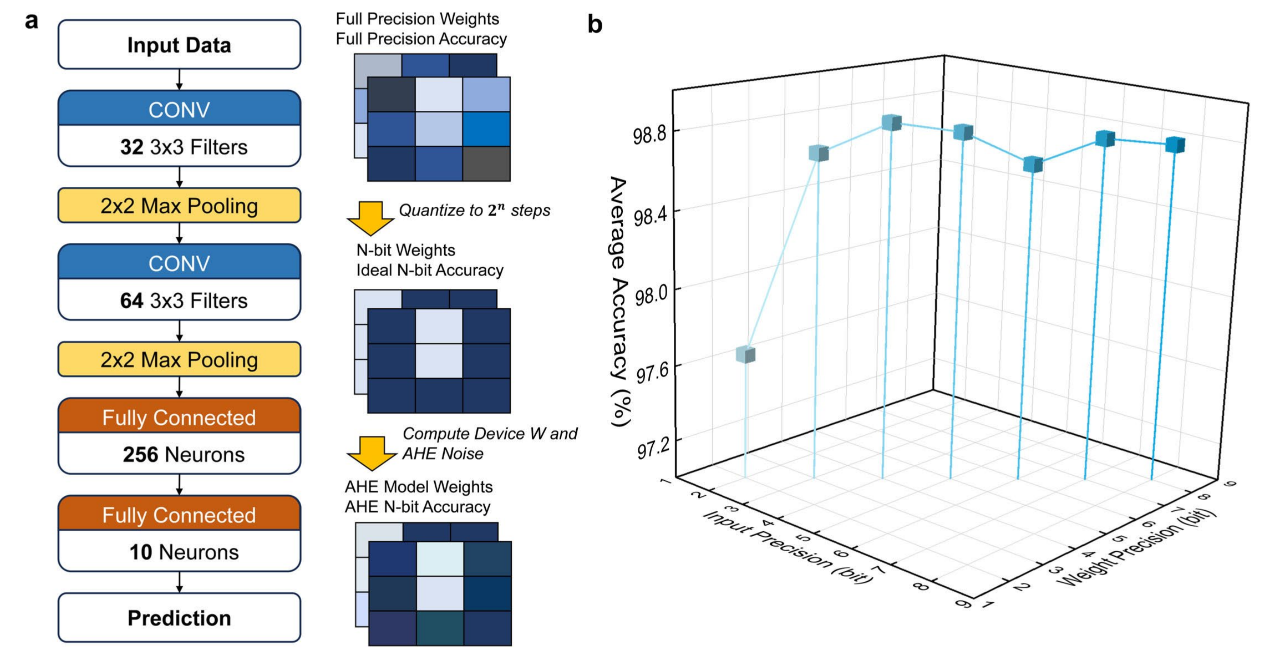

Supplementary Figure 10 | CNN flow and recognition accuracy testing. (a) Block diagram illustrating the CNN implementation flow. **(b)** Average recognition accuracy on the MNIST handwritten digit dataset as a function of input and weight precision.

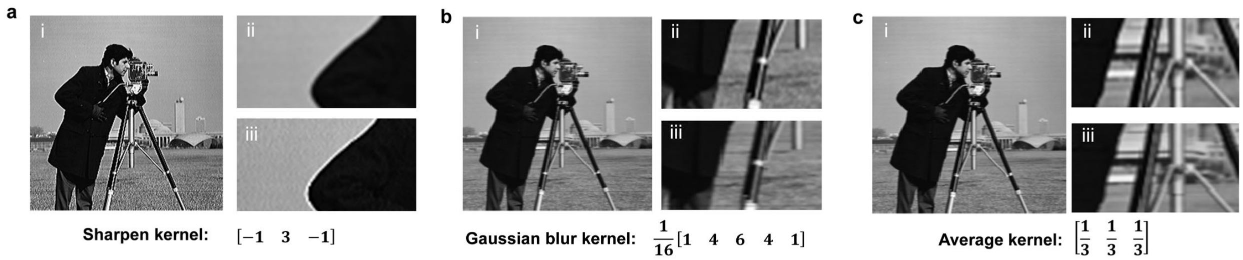

Supplementary Figure 11 | Image sharpening, Gaussian blurring, and averaging using MCA devices with different kernels. (a)(i) Sharpened 'Cameraman' image generated by an MCA device. **(b)(i)** Gaussian-blurred 'Cameraman' image generated by an MCA device. **(c)(i)** Averaged 'Cameraman' image generated by an MCA device. The convolution kernels used are shown at the bottom of each image. **(a)(ii), (b)(ii), (c)(ii)** Zoom-in views of the original 'Cameraman' image at selected regions. **(a)(iii), (b)(iii), (c)(iii)** Corresponding zoom-in views of the sharpened, blurred, and averaged images generated by MCA devices in the same regions.

Supplementary Note 1

We have developed a fast Hall measurement technique and experimentally determined the domain-wall shifting velocity.

In this setup, the Hall voltage is recorded using a high-speed oscilloscope with a detection bandwidth in the MHz range, allowing real-time tracking of sub-10 m/s domain-wall motion. For higher velocities (on the order of hundreds of m/s), GHz-level detection would be required, which can be achieved by integrating a high-frequency detection circuit, as demonstrated in Reference [1].

The measurement principle is detailed as follows. It relies on the linear dependence of the Hall voltage (V) on the domain length (L_D) within the Hall channel. As illustrated in Supplementary Fig. 12a, V is expressed as:

$$V = V_{AHE}(2\frac{L_D}{L_P} - 1) \tag{1}$$

where L_P is the Hall channel length, and V_{AHE} is the saturated anomalous Hall effect (AHE) voltage.

When a current drives the domain wall, L_D evolves with time as $L_D = v_{DW} \times t$, giving:

$$V = V_{AHE}(2\frac{v_{DW} \cdot t}{L_P} - 1) \tag{2}$$

Hence, the domain-wall velocity (v_{DW}) can be extracted from the slope of the $V - t$ curve.

Using this method, we measured the domain-wall velocity in our MCA device (Supplementary Fig. 12b). The real-time Hall Voltage vs. Time curve is shown in Supplementary Fig. 12c (right Y-axis), and the corresponding domain length (L_D) derived from Eq. (1) is plotted on the left Y-

axis. Linear fitting of $L_D - t$ yields a slope of 0.0625 m/s, representing the domain-wall velocity. The dependence of velocity on current density is summarized in Supplementary Fig. 12d.

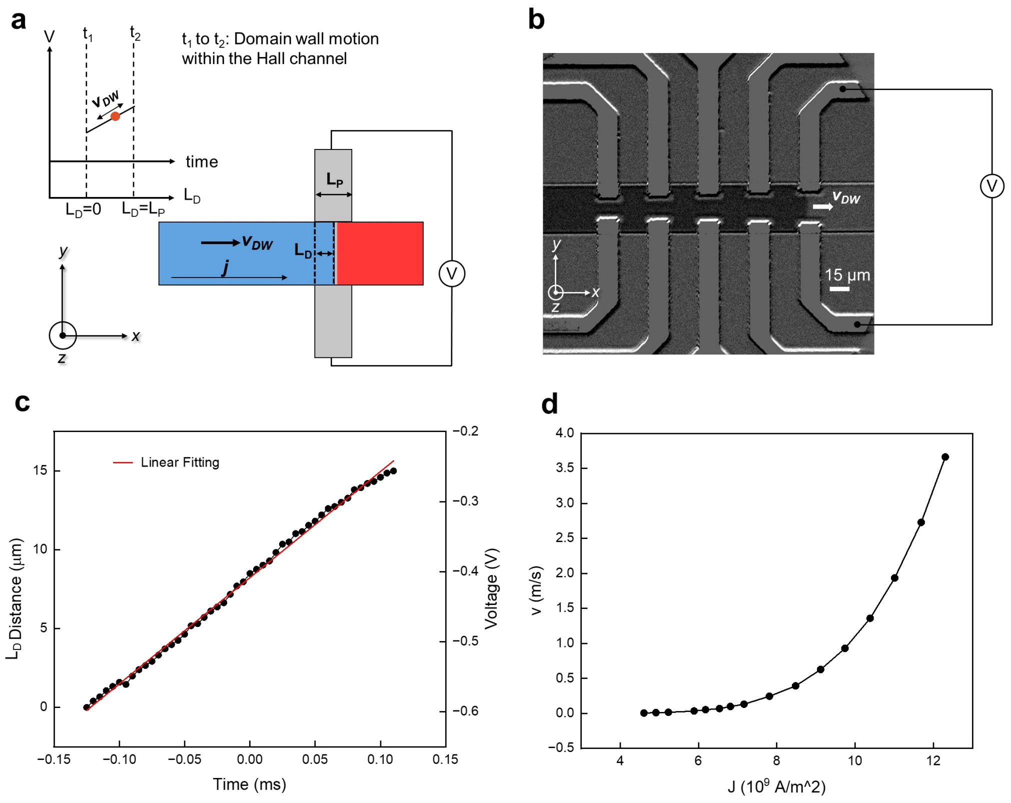

Supplementary Figure 12 | Domain wall motion speed measurement. (a), Blue rectangle represents the $-M_z$ domain, red the $+M_z$ domain, and grey the Hall channel. L_P: Hall channel length. L_D: Length of the $-M_z$ domain inside the Hall channel. A current j is applied to drive the domain wall motion. The voltage V of the Hall channel is measured in real time. Top left picture illustrates the ideal Voltage vs. Time plot for the domain wall motion. **(b),** Current-driven domain wall motion in the MCA device. Horizontal rectangle is the magnetic strip. Black color is the $-M_z$ domain, and grey the $+M_z$ domain. Pairs of vertical strips are Hall channels. **(c),** L_D and Hall Voltage vs. Time plot measured in the rightmost Hall channel. The velocity is obtained by linear fitting. **(d),** Domain-wall velocity as a function of current density.

Supplementary Note 2

We performed experiments to characterize the heating effect induced by the SOT pulse.

First, we measured the R_{xx}–T curve (device longitudinal resistance vs. temperature) for the same devices used in the manuscript. The setup is shown in Supplementary Fig. 13a. A 1 μA reading current (chosen to minimize self-heating) was applied to the current-shifting channel, and the voltage was measured across the two Hall channels using the standard four-probe method. The resulting R_{xx}–T curve (Supplementary Fig. 13b) shows a resistance of 349.72 Ω at 300 K with a slope of –0.069 Ω/K, which serves as our calibration reference. The temperature rise (ΔT) associated with Joule heating during domain shifting can be obtained from the measured change in longitudinal resistance (ΔR_{xx}) using: $\Delta T = -\frac{1\text{K}}{0.069\Omega}\Delta R_{xx}$.

To quantify the heating effect, we applied DC currents ranging from 1 μA to 1 mA and measured the equilibrium R_{xx} (Supplementary Fig. 13c). For a 1 mA current—the shifting current used in the manuscript—we obtain: $\Delta T = \frac{1\text{K}}{0.069\Omega}\Delta R_{xx} \rightarrow \Delta T = -\frac{1\text{K}}{0.069\Omega} \times (348.33\Omega - 349.72\Omega) \approx 20\ K$. This indicates that the device temperature increases from **300 K to approximately 320 K during 1mA DC current application**.

A temperature rise of ~20 K is modest in magnetic device applications and is unlikely to cause irreversible changes in magnetic properties. Larger temperature change is commonly observed in spin-orbit torque and domain-wall motion studies, without performance degradation.

Moreover, in practical operation, the device is driven by pulse with a 50% duty cycle (or lower), which further reduces the effective heating. Therefore, Joule heating is not expected to notably affect the reliability or reproducibility of our device.

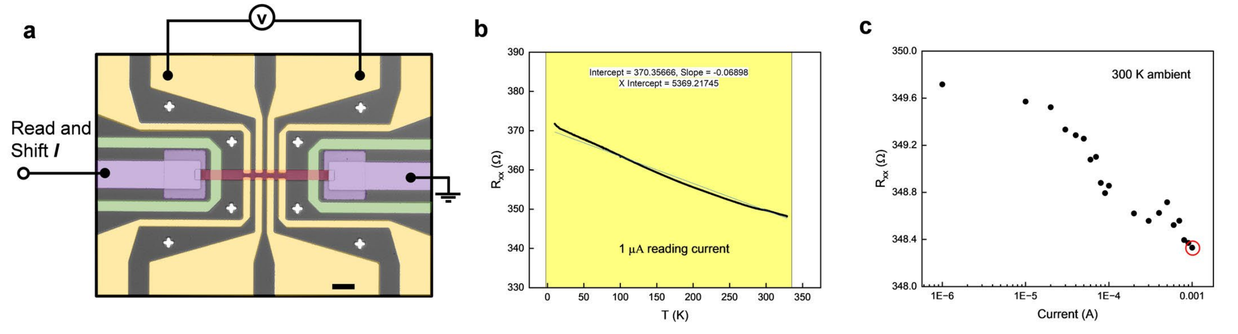

Supplementary Figure 13 | Heating effect during Shifting operation. (a), Measurement setup. **(b),** R_{xx} vs. T curve. **(c),** R_{xx} as a function of DC current amplitude. Each current is applied for 4 hours, and measurements are performed at room temperature.

Supplementary Note 3

We used MOKE imaging to quantitatively assess domain-wall synchronization during propagation.

As illustrated in Supplementary Fig. 14a, a domain is bounded by two domain walls (DWs), and their spacing—the domain length (L_D)—may fluctuate during motion. Thus, the degree of synchronization can be evaluated by measuring the variation in L_D as the domain moves along the track.

We experimentally prepared two representative cases: a wide domain and a narrow domain. The result is shown in Supplementary Figs. 14b & c. For wide-domain propagation, the measured domain lengths exhibit a standard deviation of **0.15 µm (1.9%)**, and for narrow domains, **0.10 µm (2.1%)**. These results confirm the high stability of domain-wall motion.

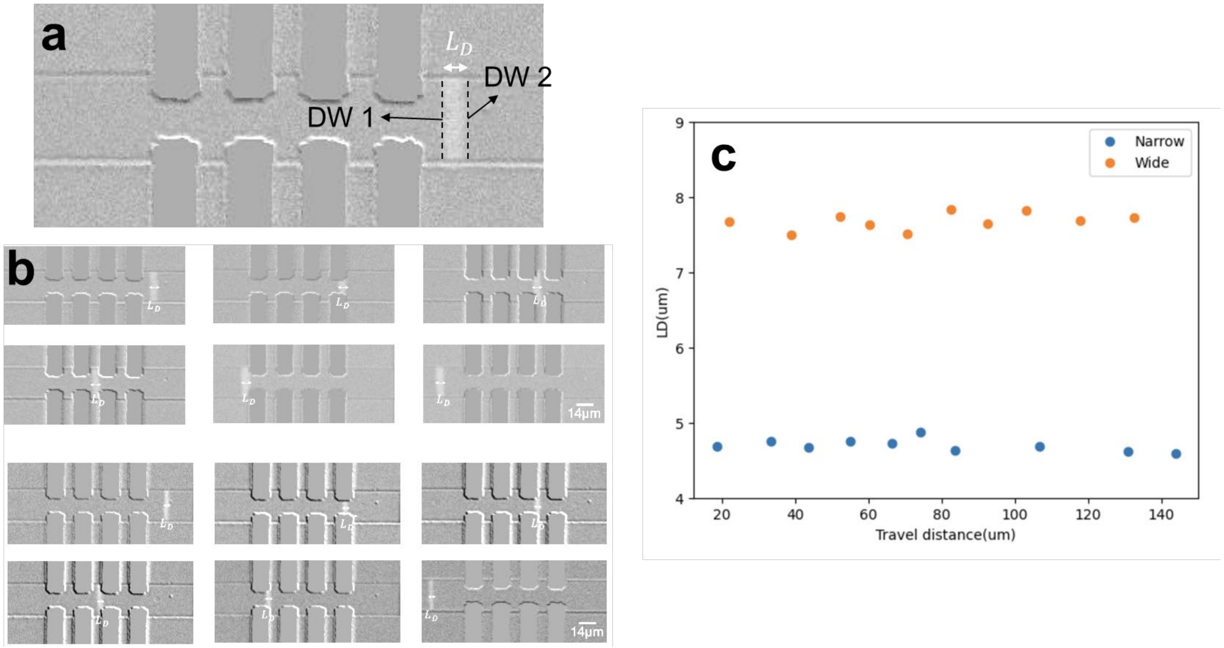

Supplementary Figure 14 | Domain wall synchronization test. (a), MOKE image of a domain. It consists of two domain walls, DW 1 and DW 2. **(b),** Wide (Top) and narrow (Bottom) domain

propagation across MCA device. **(c),** Domain length vs. Travel Distance.

Supplementary Note 4

We performed micromagnetic simulations to investigate the influence of domain-wall pinning in downscaled devices.

The simulation is performed in devices with dimensions of **$256 \times 64 \times 1$ nm³** and **$1024 \times 256 \times 1$ nm³** (Supplementary Fig. 15ai). Following established methods [2,3], pinning was modeled by introducing grains, as shown in Supplementary Fig. 15aii. The grain size and associated material parameters—including perpendicular magnetic anisotropy, saturation magnetization, and Dzyaloshinskii–Moriya interaction—were assigned Gaussian distributions to emulate realistic disorder.

We then examined SOT-driven domain-wall motion under varying current densities. The influence of pinning is reflected in the velocity–current density (v–J) characteristics (Supplementary Fig. 15b). In both device sizes, we observe depinning and creep regimes—hallmarks of pinning-limited motion. The smaller device exhibits a higher depinning threshold current, indicating stronger pinning effects due to the reduced dimensions. Once the depinning transition is surpassed, the motion enters the flow regime, where the dynamics become largely independent of pinning [4]. This flow regime represents the desirable operational state for domain-wall motion devices.

In conclusion, **device downscaling increases the required current density to achieve a given domain-wall velocity**.

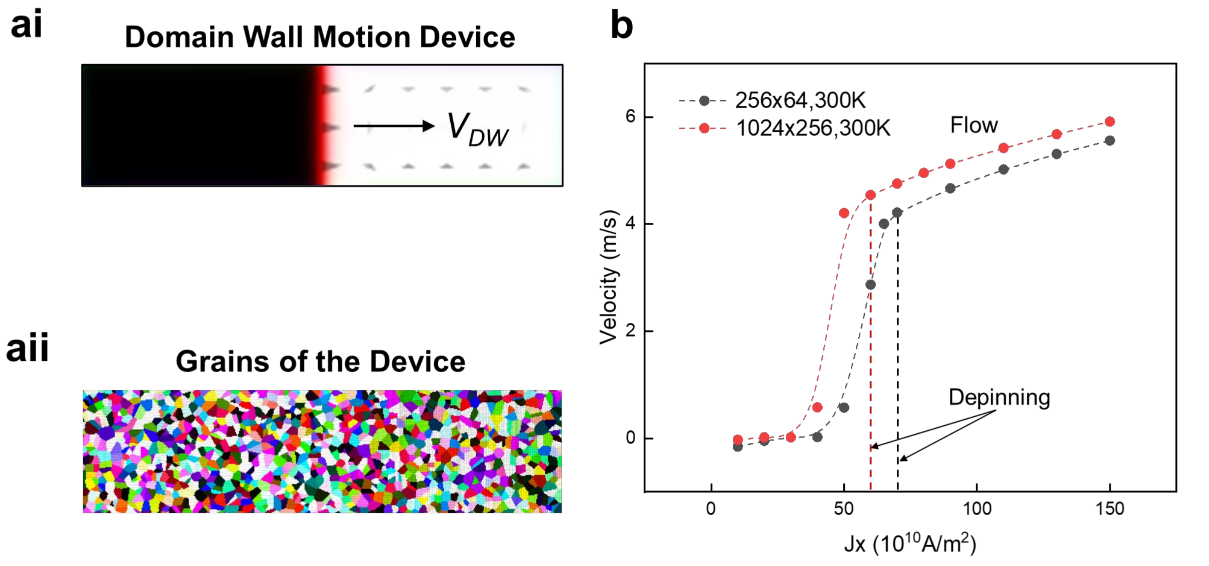

Supplementary Figure 15 | Influence of domain-wall pinning in downscaled devices. (ai), Domain wall motion device. Black: -Mz, White: +Mz, Red: Domain Wall region. **(aii),** The device is divided into grains with Gaussian distributed size and material properties. Different color represents different perpendicular magnetic anisotropy values. **(b),** Velocity vs. Current Density plot.

Supplementary Note 5

The device yield is investigated.

An optical image of the fabricated sample is shown in Supplementary Fig. 16a, containing 354 MCA devices. A magnified view of the top block and a single representative device are shown in Supplementary Figs. 16b and 16c, respectively.

To qualify as functional, a device must successfully perform the Write, Shift, and Read operations. Across the entire project, we have measured 64 MCA devices, of which 58 were fully operational, corresponding to a yield of **~90.6%**, primarily limited by the Shift operation.

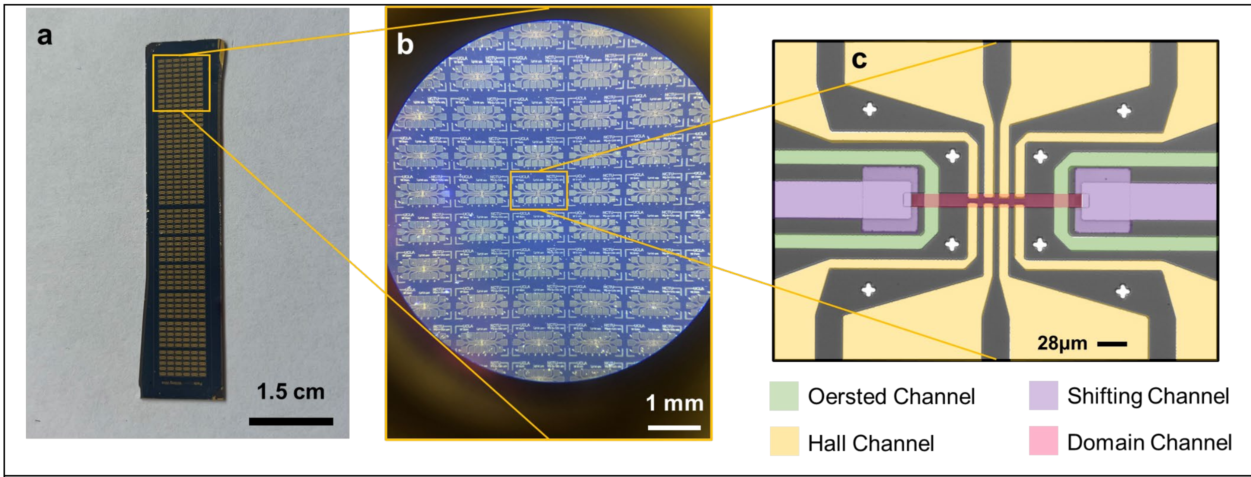

Supplementary Figure 16 | Sample and device. (a), An actual fabricated sample. **(b),** A block of the sample. **(c),** A single MCA device from (b).

(a) Write

Domain nucleation and length control are achieved using an Oersted-field line, as described in the Main Text. The mechanism and fabrication requirements are both simple and robust. The writing lines are tens of micrometers in width, easily handled by standard lithography. No failures were observed in the Write operation, giving a **100% yield**.

(b) Shift

The main source of yield loss arises from local fabrication defects that hinder domain-wall motion. A representative defective device is shown in Supplementary Fig. 17a, with the magnified region in Supplementary Fig. 17b highlighting structural imperfections. Such defects reduce domain-wall velocity, requiring higher current densities to achieve the same speed and thereby increasing energy consumption.

For quantitative evaluation, we measured domain-wall velocities in all 64 devices under the same driving current density of 8.33×10^9 A/m^2 (the value used in the Main Text). The results, summarized in Supplementary Fig. 17c, show that typical devices exhibit velocities around 0.5 m/s, while defective ones fall below 0.4 m/s. We therefore define devices with $v < 0.4$m/s as non-functional. The resulting yield is **58/64 (~90.6%)**.

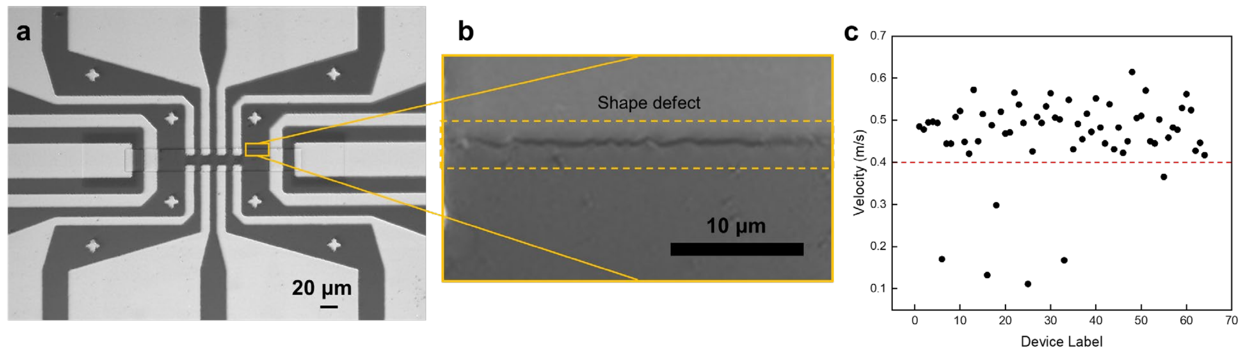

Supplementary Figure 17 | Device yield from the shifting operation. (a), Microscope image of an MCA device with defects. **(b),** Zoom-in image of **(a)** showing the shape defect in magnetic strip. **(c),** Device variation in domain motion velocity.

(c) Read

The Read operation relies on detecting the anomalous Hall effect (AHE), which scales linearly with the width W_P. AHE sensing is highly robust, with only small deviations from ideal linearity

due to fabrication non-idealities. As demonstrated in the Main Text, the readout quality remains sufficient for algorithmic implementation. Thus, the Read operation also shows a **100% yield**.

In summary, the domain-wall motion (Shift) stage represents the primary yield bottleneck. Among 64 tested devices, 58 functioned correctly, **yielding ~90.6% overall,** as summarized in Supplementary Table 1.

Supplementary Table 1 | Device yield summary.

Yield	Write Success Rate	Shift Success Rate	Read Success Rate
90.6%	100%	90.6%	100%

Supplementary Note 6

The device lifetime (endurance) is investigated.

The endurance can be divided into two parts, magnetic and non-magnetic. The magnetic endurance is essentially unlimited, since magnetization reversal itself is inherently non-destructive, it has no wear-out mechanism — as emphasized in fundamental reviews of spintronics and MRAM technology [5-7]. The non-magnetic endurance (mostly electric endurance) — including tunnel barrier reliability, interface degradation, dielectric breakdown, and electrical stress — has been experimentally demonstrated to exceed 10^14 switching cycles at 90% duty cycle without degradation [8,9].

To experimentally assess our own device stability, we monitored the anomalous Hall effect (AHE) signal under continuous operation. Using the same device from the heating effect test, we applied a 1 mA DC current (domain-motion current) and repeatedly measured the AHE loops over five days. More than 1000 AHE loops were recorded (Supplementary Fig. 18a). All curves overlap nearly perfectly, indicating no measurable change in either magnetic (AHE resistance, PMA, DMI, Ms) or electrical (longitudinal resistance, contact resistance) properties.

Assuming a 2 ns operation pulse and 50% duty cycle, this corresponds to a projected endurance of **2.16×10^14 operation,** which is sufficient for most AI and signal-processing workloads. A longer test duration would likely confirm even higher endurance. A zoom-in of the coercive-field region (Supplementary Fig. 18b) shows minor fluctuations (~0.3 Oe), attributable to expected thermal noise near coercivity.

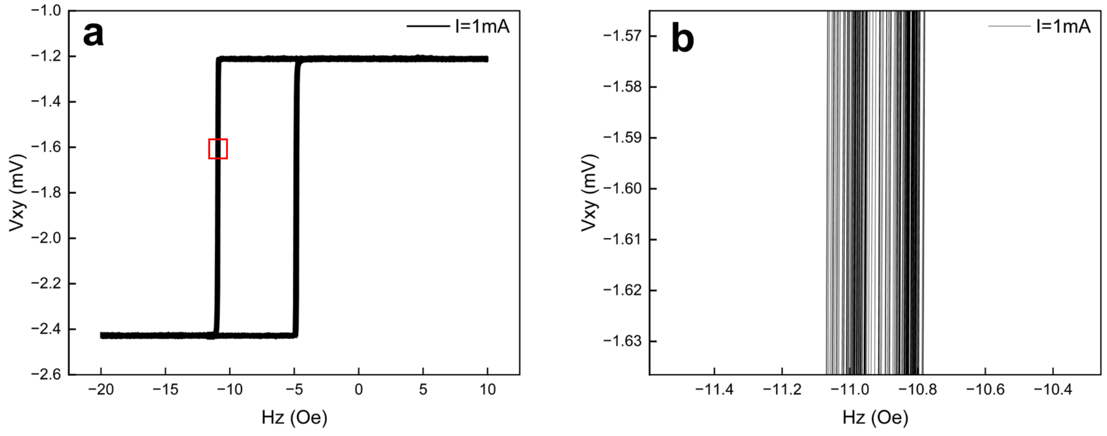

Supplementary Figure 18 | Endurance test for the MCA device. (a), 5-day AHE measurement. Offset in Hz is due to the remanence field of the electro-magnet. **(b),** Zoom-in plot of the red box region of (a), at the coercivity.

Supplementary Note 7

The influence of W_P variation on short-time Fourier transform (STFT) result is investigated.

In Fig. 3c of the Main Text, the device is used to perform the STFT. The designed W_P sequence for this device is 6–8–6–8–6–8–6 μm, as labeled in Supplementary Fig. 19a(i). The corresponding measured V_{AHE} values are shown in Supplementary Fig. 19a(ii). The $V_{AHE} - W_P$ dependence confirms the intended mapping between W_P and the convolution kernel coefficients.

Although small variations exist among the measured V_{AHE} values, this level of precision is sufficient for algorithmic implementation. As demonstrated in Fig. 3 of the Main Text, the Fourier transform was performed using the raw data from Supplementary Fig. 19a(ii), yielding accurate reconstruction of the Fourier amplitudes (Supplementary Fig. 19b(i); corresponding to Fig. 3c(iv) in the Main Text). A zoomed-in view of the boxed region [Supplementary Fig. 19b(ii)] shows only small fluctuations due to fabrication variations in W_P, which do not affect the overall transform accuracy.

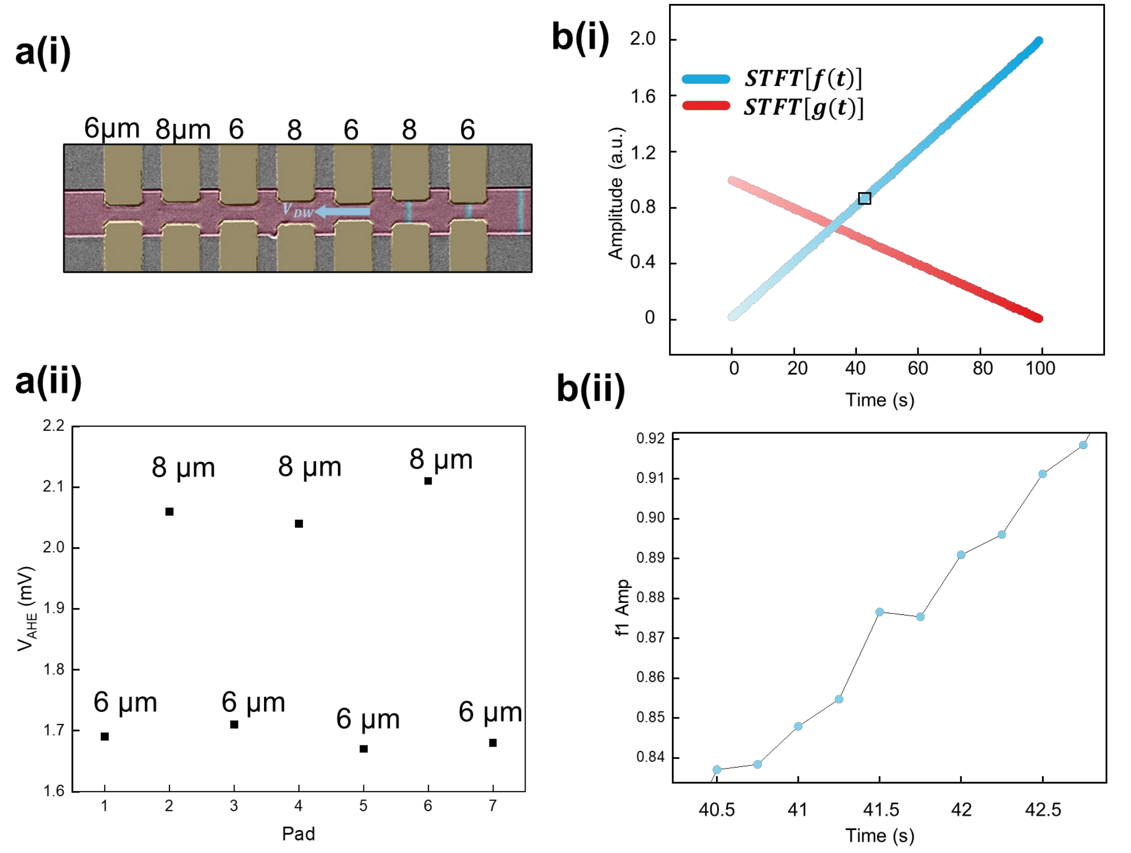

Supplementary Figure 19 | Mapping of convolution kernel and Fourier transform result. (ai), Fig. 3c device microscope image. This device is designed to have two W_P, 6 μm and 8 μm. **(aii),** Measured V_{AHE} of the 7 W_P. **(bi),** Fourier transformed result. **(bii),** Zoom-in plot of the black box region in (bi).

Supplementary Note 8

To further validate the practical potential of our device, we extended our evaluation beyond the MNIST dataset to the more challenging CIFAR-10 image classification task.

Compared with MNIST, CIFAR-10 images have a higher dimensionality (**32 × 32 × 3**) and involve convolutional layers with approximately **20×** more kernel weights, thereby providing a more stringent benchmark for computational performance.

In our experiments, we incorporated readout deviations originating from both domain length and weight variability. Representative CIFAR-10 images with and without domain-length deviation are shown in Supplementary Fig. 20a, illustrating that the induced variation has a negligible visual impact—an important prerequisite for maintaining correct classification performance.

The classification results obtained from our MCA device are presented in Supplementary Fig. 20b. The achieved recognition accuracy is approximately **80%**, which is comparable to state-of-the-art CIFAR-10 baselines (see Ref. [10]). These results confirm that our MCA device sustains robust performance even for more complex datasets beyond MNIST.

We note that the current accuracy limit (~80%) primarily arises from the simplified neural network architecture used for this demonstration rather than any intrinsic device limitation. Further improvements can be expected by employing more advanced network structures—such as ResNet—which are beyond the scope of the present work.

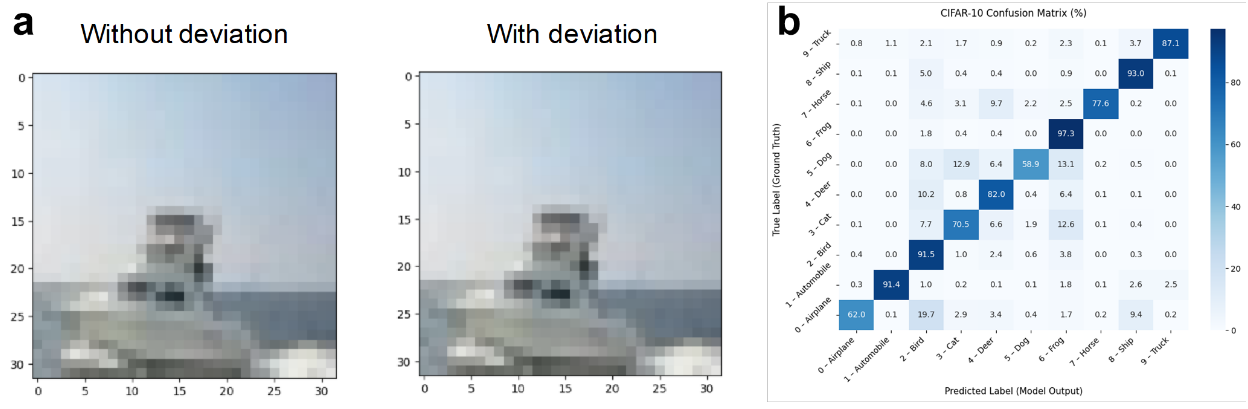

Supplementary Figure 20 | CIFAR-10. (a), CIFAR-10 image without and with domain length deviations. **(b),** CIFAR-10 recognition matrix from our device. For example, when input is an Automobile image, we have 91.4% chance to recognize it as an Automobile. An average of ~80% accuracy is achieved.

Supplementary Note 9

The effect of nonlinear deviations in electrode spacing (W_P) and domain length (L_D) are investigated.

In our experimental setup, the nonlinear deviations mainly come from two sources: (1) variations in the domain length (input variation) and (2) variations in the Hall pad width (weight variation).

The nonlinear deviations of these two factors are presented in Main Text Figs. 2f and 2h. Quantitatively, the average deviation in Hall pad width is approximately **7%**, while the variation in domain length is about **2%**.

The observed variations introduce small errors in the convolution result, which could translate into algorithmic deviations. To evaluate their impact, we performed additional experiments as described below.

(a) Input variation

We first examined the effect of input variation by comparing the handwritten-digit dataset processed using the raw experimental data from Fig. 2h (which includes domain-length deviations) with the ideal case. As shown in Supplementary Fig. 21, the images with and without domain-length variation appear visually identical, as the experimental domain-length fluctuation is only ~2%. This confirms that the essential image features required for accurate recognition are well preserved.

The corresponding handwritten-digit recognition task, incorporating these nonlinear deviations, achieves an accuracy of **98.94%**, demonstrating that domain-length variation has a negligible effect on the algorithm's performance.

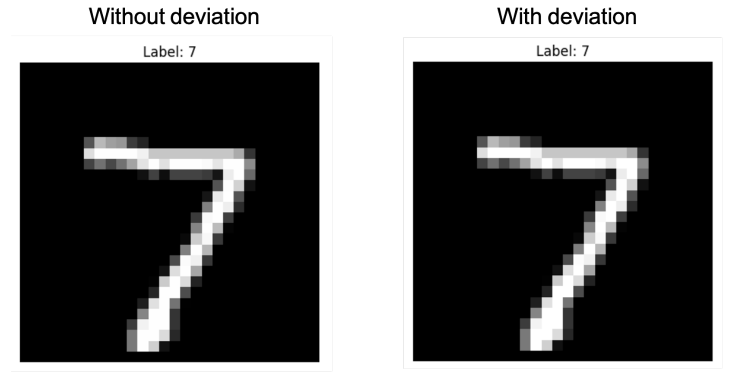

Supplementary Figure 21 | Input with and without deviation. Left: MNIST image without incorporating domain variation. **Right:** MNIST image with domain length variation. Both images show a clear digit shape and the difference is negligible.

(b) Weight variation

In the Main Text, we already accounted for variations in the weights by directly using the raw V_{AHE} readouts from different Hall channels as the weight values.

As discussed in the Main Text, the training process was performed in software, whereas the MCA device is designed for inference with fixed weights. Accordingly, the kernel values derived from the training results are approximated using the nearest experimental V_{AHE} values, as illustrated below in Supplementary Table 2 and Supplementary Table 3:

Ideal convolution kernel (training):

Supplementary Table 2 | Ideal convolution kernel.

0.1399	-0.289	0.172
0.489	0.251	-0.521
0.2173	-0.299	-0.452

Raw V_{AHE} approximation (experiment):

Supplementary Table 3 | Experimental approximation.

0.1400	-0.271	0.184
0.490	0.239	-0.524
0.2208	-0.305	-0.478

We then performed inference using both **experimental input** and **weight variations** (raw V_{AHE} values incorporating domain-length and Hall-pad deviations). The resulting recognition accuracy remains high at **98.80%**, confirming that the observed nonlinearities do not significantly impact the algorithm's performance.

Supplementary Note 10

The weight (kernel coefficients) are **dynamically generated and tunable** in a defined time sequence.

As illustrated in Supplementary Fig. 22, the kernel coefficients are encoded in the upper magnetic strip. Similar to the domain generation method used in the Hall-based MCA, the magnetic domains are nucleated and tuned using current-induced Oersted fields generated by a local Oersted channel.

The sequence proceeds as follows: the first domain is generated and its width (corresponding to the coefficient value) is adjusted (Supplementary Fig. 22a); it is then shifted to the first MTJ junction region (Supplementary Fig. 22b). The second domain is subsequently generated (Supplementary Fig. 22c), after which both domains are shifted together into their designated MTJ junctions, and the third domain is generated (Supplementary Fig. 22d). This process continues until all kernel-coefficient domains are deployed. When a new set of kernel coefficients is required, the same procedure can be repeated to redefine the domain pattern.

Thus, the kernel domains are generated sequentially, and they remain **tunable and movable** within the MTJ array.

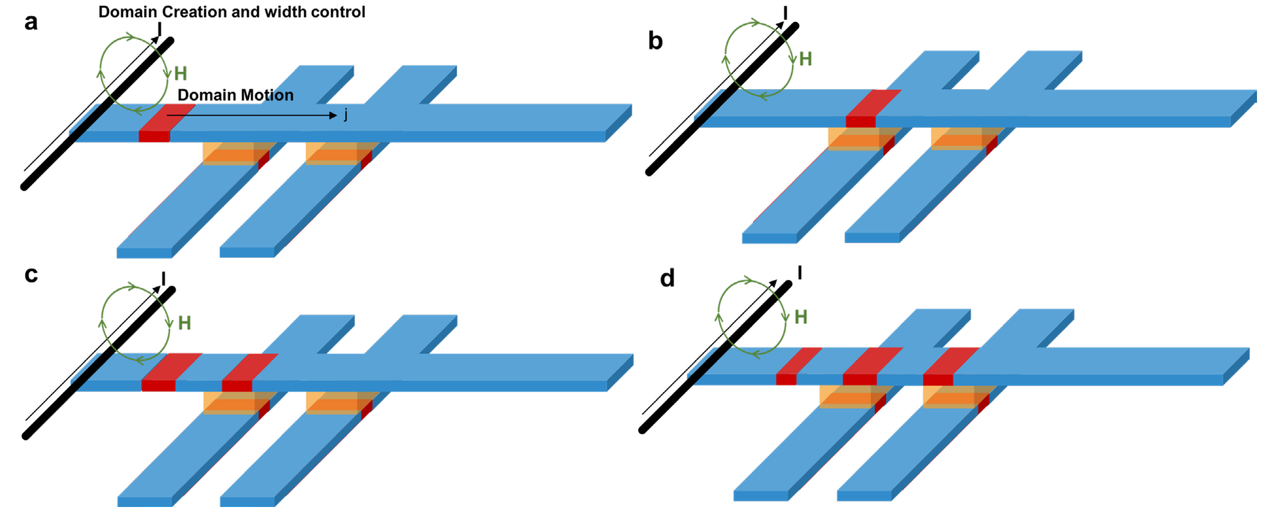

Supplementary Figure 22 | Kernel coefficient domain generation process. a, The first domain is created and tune by Oersted field. **b,** The first domain is shifted to the MTJ junction region. **c,** The second domain is generated and tuned. **d,** The first and second domain is shifted to the MTJ junction region. After this, the third domain is generated.

Supplementary Note 11

The magnetic domains in the upper (kernel) and lower (input) strips can be controlled independently, as illustrated in Supplementary Fig. 23. The upper-strip domains are generated as described above, while the lower-strip domains (input data) are produced using the same sequential domain-generation and shifting approach. Independent Oersted field lines and current channels allow precise temporal and spatial control of both layers, ensuring synchronized alignment of input and kernel domains within each MTJ junction.

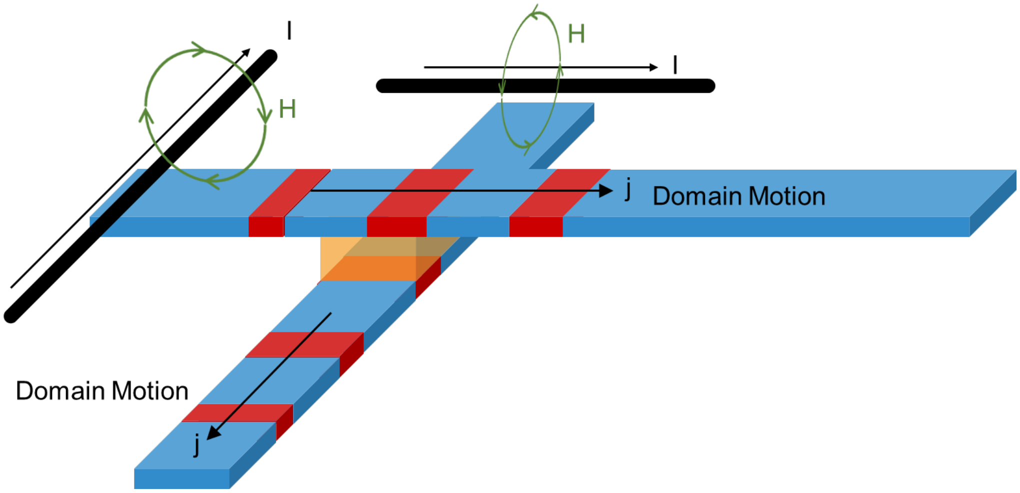

Supplementary Figure 23 | Simultaneous control of upper and lower magnetic domains.

Supplementary Note 12

The red, blue, and purple squares in Fig. 4aiii coexist simultaneously in the crossing area. To clarify this, we have redrawn Fig. 4aiii as Supplementary Fig. 24, following the same color code and the definitions of L_{DX}, L_{DY}, and W.

The top and bottom magnetic strips overlap in the junction region (crossing area), as illustrated in Supplementary Fig. 24a(i). Within this region, each magnetic layer contains two domains, represented by red and blue rectangles in Supplementary Fig. 24a(ii). As shown in Supplementary Fig. 24b, four possible domain configurations can exist in the overlapping area:

1. Region ①: Top:-z, Bottom:-z
2. Region ②: Top:+z, Bottom:-z
3. Region ③: Top:+z, Bottom:+z
4. Region ④: Top:-z, Bottom:+z

Here, the **red** squares correspond to overlapping (–z, –z) domains (region ①), the **blue** squares to overlapping (+z, +z) domains (region ③), and the **purple** squares to (+z, –z) or (–z, +z) domains (region ② ④).

In summary, the red, blue, and purple regions represent the **coexisting domain configurations** of the top and bottom layers within the crossing area. They collectively describe one possible magnetic state; different configurations can occur if the domain positions or lengths (L_{DX}, L_{DY}) are varied—for example, when the top domain becomes entirely –z in the overlapping region.

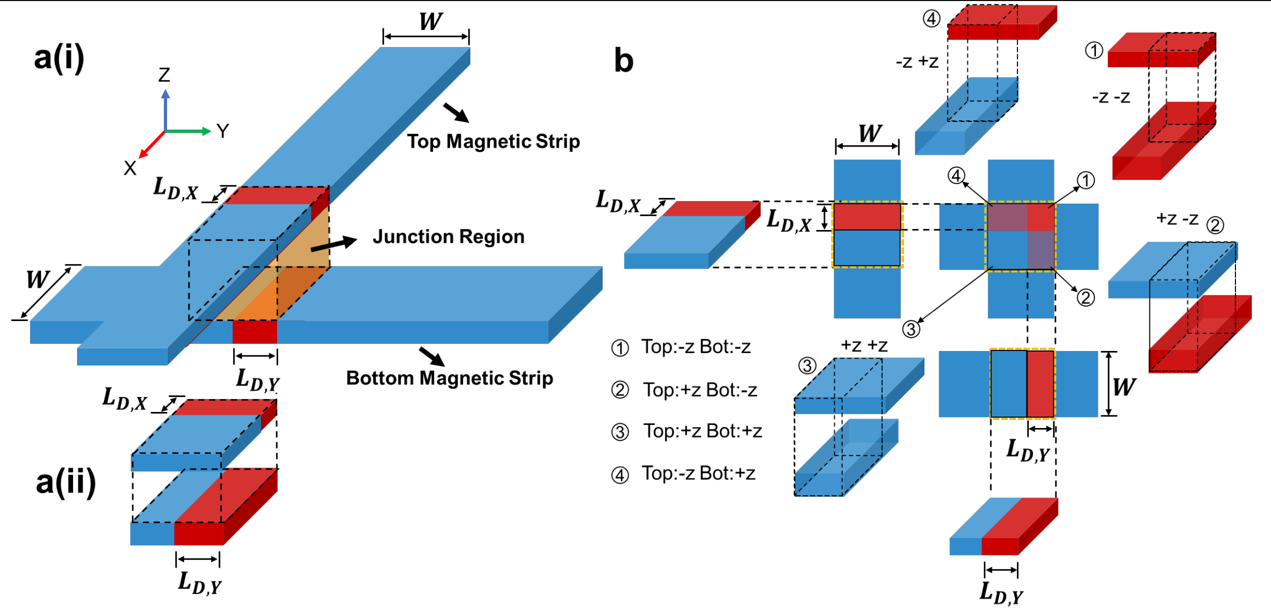

Supplementary Figure 24 | Schematic of the MTJ-MCA. (ai), The MTJ-MCA schematic. Blue: +z domain. Red: -z domain. Yellow: Junction region (crossing area). Strip width: W. Top -z domain length: $L_{D,X}$. Bottom -z domain length: $L_{D,Y}$. **(aii),** Top and bottom domain configuration at the crossing area. **(b),** Detailed view of the crossing area. It can be divided into four regions. Red square (region ①): Top:-z Bottom:-z; Purple square (region ②): Top:+z Bottom:-z; Blue square (region ③): Top:+z Bottom:+z; Purple square (region ④): Top:-z Bottom:+z. The corresponding 3D view are shown.

Supplementary Note 13

Calculation of Area, Throughput, and Energy of the MCA Versus Standard CMOS

We provide a detailed benchmarking of the Magnetic Convolutional Accelerator (MCA) relative to conventional CMOS technology. Our evaluation focuses on three aspects: area, throughput, and energy consumption, using an 8-bit, 1024-point convolution operation as a representative case under a 28 nm technology node.

Device size constraints

The minimum achievable dimensions of each convolution unit play a critical role in determining the total area and computational throughput of the system. The physical size is constrained by three main factors:

(1) Lithographic patterning limits and fabrication granularity,

(2) Stability of nucleated domains against thermal fluctuations,

(3) Resolution requirements to support input and weight quantization.

Each of these factors is analyzed in detail below.

(1) Lithography and fabrication granularity

In a standard 28 nm CMOS process, the minimum feature size that can be patterned reliably is approximately 30 nm, with a typical fabrication granularity of 1 nm for dimensional increments.

(2) Domain stability under thermal fluctuations

The free energy (E) required to form a magnetic domain arises from contributions from the domain wall energy, the demagnetization field, and any external applied field. It can be expressed as:

$$E = 2W_D\sigma t - \mu_0 {M_s}^2 W_D L_D t - 2\mu_0 H_C M_s W_D L_D t \quad (1)$$

where W_D and L_D are the domain width and length, t is the magnetic film thickness, σ is the domain wall energy density, and H_C is the coercive field.

Stable domain formation requires that the free energy decreases with domain width:

$$\frac{\partial E}{\partial W_D} < 0$$

Additionally, the energy barrier must be large enough to resist thermal perturbations:

$$\frac{E}{K_B T} > \Delta \quad (2)$$

where K_B is the Boltzmann constant, T is the absolute temperature, and Δ is the required thermal stability factor, typically set to 30 for logic and 40 for memory applications.

Using the material parameters of the Ta/CoFeB/MgO stack employed in this work (summarized in Supplementary Table 4), the minimum domain length is calculated to be approximately 8.44 nm.

Supplementary Table 4 | Device and material parameters for a Ta/CoFeB/MgO system.

Symbol	Definition	Value
σ	Domain Wall Energy Density	$5.3427\ mJ/m^2$
t	Ferromagnet film thickness	$1\ nm$
M_s	Saturation Magnetization	$10^6\ A/m$
H_C	Magnetic Coercivity	$3.98 \times 10^3\ A/m$
δ	DW Motion Speed	$26\ m/s$
J_{Shift}	AHE DW Current Density	$1.1 \times 10^{12}\ A/m$

J_{Shift}	MTJ DW Current Density	$10^9\ A/m$
J_{Hall}	Hall Reading Current Density	$8.33 \times 10^8\ A/m$
I_{TMR}	Junction Reading Current	$1\ \mu A$
l_{min}	Minimum Domain Length	$8.44\ nm$

(3) Resolution requirements for input and weight encoding

For n-bit input and weight precision, the device must support fine adjustments to domain lengths and Hall electrode spacings. The total dynamic range for domain lengths is:

$$l_{min} + 2^n \Delta l$$

And for Hall spacings:

$$w_{min} + 2^n \Delta w$$

where Δl and Δw represent the minimum increments in domain length and electrode spacing, respectively.

In devices where domains are created by pulsed currents, the domain length resolution Δl depends on the domain wall velocity δ and the minimum current pulse duration p_{min} according to:

$$\Delta l = \delta p_{min} \tag{3}$$

In 28 nm technology, p_{min} is approximately 10 ps, corresponding to a Δl of about 0.26 nm. Meanwhile, Δw is determined by the lithographic granularity, typically 1 nm.

Based on these criteria, the optimal device dimensions to support 8-bit resolution are:

- Hall-MCA: $L_P = 75\ nm, W_P^{max} = 346\ nm$
- MTJ-MCA: $L_P = 75\ nm, W_P^{max} = 75\ nm$

The area (A) for a full 8-bit, 1024-point MCA array is given by:

$$\left((W_P^{max} + F) \times (L_P + F) + 100F^2\right) \times 1024 \quad (4)$$

where F is the minimum feature size (30 nm).

Using this formula, the estimated areas are:

- 133 μm² for the Hall-based MCA
- 103 μm² for the MTJ-based MCA.

Operating Speed Analysis

The performance speed of the MCA is fundamentally limited by the slower of two sequential phases:

(1) Domain writing and shifting, and

(2) Electrical signal sampling and accumulation (via AHE) or tunneling magnetoresistance (TMR)).

(1) Domain Nucleation and Shifting Delay:

The first phase delay is determined by the time required for a DW to traverse the entire distance corresponding to a single convolution unit, including the minimal feature pitch (F). This delay is calculated as $(L_P + F)/\,\delta$, where δ denotes the DW velocity. For the device configurations analyzed, this leads to a delay of approximately $t_d = 4.04\ ns$.

(2) Signal Sampling and Summation Delay:

The second phase consists of two steps: (i) charging the sampling capacitors, and (ii) summing the accumulated charges via switched-capacitor circuits.

- The sampling time can be estimated using: $t_{sample} = C_{sample}V_{AHE\ (TMR)}/I_{AHE\ (TMR)}$, where C_{sample} is the sampling capacitance, $V_{AHE\ (TMR)}$ is the AHE or TMR voltage, and $I_{AHE\ (TMR)}$ is the corresponding current.
- For a typical configuration with $C_{sample} = 1$ fF, voltages of ±10 mV (AHE) or ±100 mV (TMR), and current magnitudes of ~1 μA, the capacitor charging delay is on the order of tens to hundreds of picoseconds.
- The summation process, governed by CMOS switch speeds in 28 nm technology, typically introduces an additional delay of only a few picoseconds, and is therefore negligible.

Given that domain shifting constitutes the dominant bottleneck, the MCA's throughput can be estimated as:

$$\text{Throughput} = \frac{1}{\text{Delay}} \tag{5}$$

Leading to a maximum operating frequency of approximately 248 MHz for both Hall-MCA and MTJ-MCA systems. The per-unit metrics of the MCA is summarized in Supplementary Table 5.

Supplementary Table 5 | Per-unit metrics of the MCA.

Metric	8Bit 1D MCA	8Bit 2D MCA
L_P	$75\ nm$	$75\ nm$
W_P^{max}	$346\ nm$	$75\ nm$
$R_{s(J)}$	$47.5\ \Omega$	$219\ \Omega$
F	$30\ nm$	$30\ nm$
R_{wire}	$20\ \Omega$	$20\ \Omega$
t_{cycle}	$4.04\ ns$	$4.04\ ns$
E_{read}	6.4×10^{-16} J	6.6×10^{-16} J
E_{shift}	10^{-12} J	5.96×10^{-16} J
E_{write}	3.49×10^{-15} J	3.49×10^{-15} J

Energy Consumption Analysis

The energy consumed by the MCA during operation can be categorized into two phases:

(1) Readout energy during sampling and summation, and

(2) Energy during domain writing and shifting processes.

(1) Sampling and Summation Energy:

This phase involves three main contributions:

(a1) Ohmic loss due to the readout current flowing through the magnetic strip,

(a2) Charging of the sampling capacitors, and

(a3) Switching energy associated with the sampling and summation switches.

The total readout energy per operation can be expressed as:

$$E_{read}^{AHE} = I_{Hall}^2 R_s t_{cycle} + C_{sample} V_{AHE}^2 + 4C_{sw} V_{DD}^2 \quad (6)$$

$$E_{read}^{TMR} = I_{TMR}^2 R_J t_{cycle} + C_{sample} V_{TMR}^2 + 4C_{sw} V_{DD}^2 \quad (7)$$

Where:

- $C_{sw} = C_{sample}$ is the sampling/summation switch capacitance,
- $V_{DD} = 0.8\ V$ is the supply voltage,
- I_{Hall} and I_{TMR} are the Hall and TMR readout currents, respectively,
- R_s is the strip resistance, and $R_J = 100\ K\Omega$ is the tunneling junction resistance,
- t_{cycle} is the convolution cycle period.

Using the measured and assumed parameters, this yields:

- $E_{read}^{AHE} = 6.4 \times 10^{-16}\, J$
- $E_{read}^{TMR} = 6.6 \times 10^{-16}\, J$

(2) Domain Writing and Shifting Energy:

The energy for shifting and input writing includes:

(b1) Energy for shifting existing domains to the next convolution site, and

(b2) Energy for writing (nucleating) a new domain at the strip's starting point.

These are given by:

$$E_{shift} = I_{shift}^2 R_s t_d \tag{8}$$

$$E_{write} = I_{generate}^2 R_w t_w \tag{9}$$

Where:

- I_{shift} and $I_{generate}$ are the domain shifting and nucleation currents,
- R_w is the resistance of the Oersted strip, and t_w is the pulse width for domain nucleation.

The energy to generate a new domain across the strip is calculated as

$$E_{write} = 3.49 \times 10^{-15}\, J$$

For the current Ta/CoFeB/MgO system:

- Domain shifting energy per convolution unit is $E_{shift} = 10^{-12}\, J$

- By further optimizing domain pinning for TMR-based designs, E_{shift} can be reduced to $5.96 \times 10^{-16}\, J$.

(3) Total Energy Consumption:

Considering a full 1024-point convolution, the total energy is:

$$\left(E_{read} + E_{shift} + E_{write}\right) \times 1024 \tag{10}$$

leading to final estimates:

- Hall-MCA: $1.03\ nJ$
- MTJ-MCA: $4.86\ pJ$

Supplementary Note 14

CMOS Benchmarking Methodology for Convolution Operation

To enable a fair comparison, a dedicated CMOS application-specific integrated circuit (ASIC) was designed to replicate the functionality of the MCA device—specifically, performing 8-bit, 1024-point convolutions. The architecture is illustrated in Supplementary Fig. 25.

The CMOS convolution hardware consists of:

- **8-bit shift registers** for sequential input data shifting,
- **8-bit fixed-weight registers** for storing convolution kernels,
- **Parallel 8-bit multipliers** to compute the input–weight products,
- **An 8-bit adder tree** to sum the partial products.

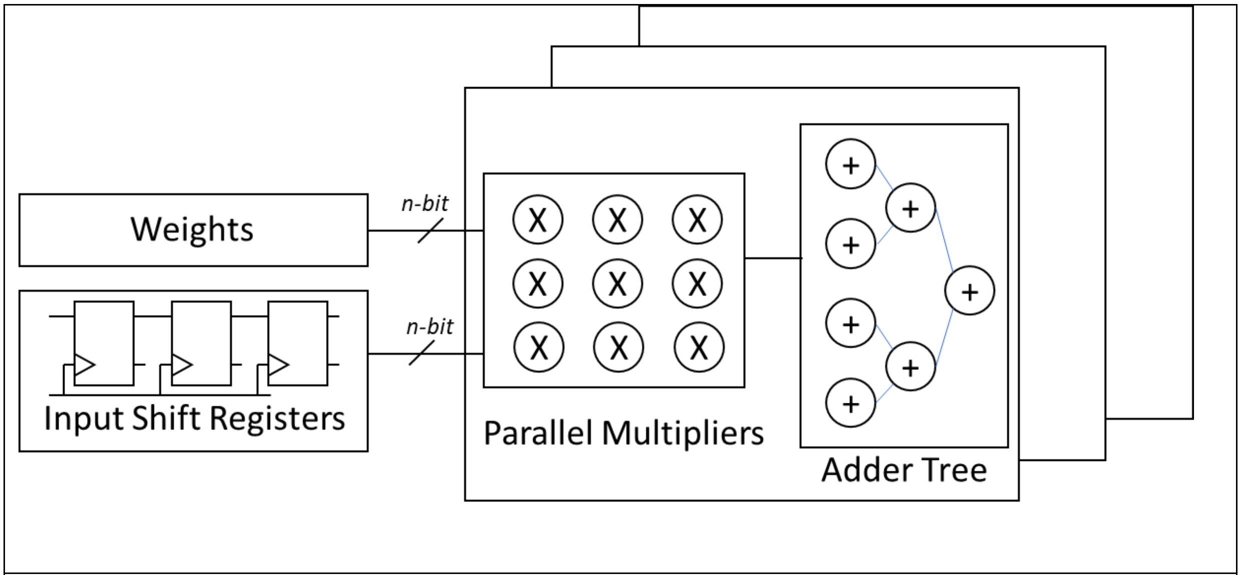

Supplementary Figure 25 | CMOS circuit architecture used for convolution benchmarking.

Each circuit component shown in Supplementary Fig. 25 was synthesized using a standard digital cell library provided by the semiconductor foundry. The library offers detailed specifications for

each logic cell, including physical layout information, delay characteristics, leakage properties, and energy consumption metrics.

The benchmarking specifications were established as follows:

- **Area Estimation:** The reported area reflects the fully synthesized layout, accounting for both the active components and routing overheads.
- **Energy Estimation:** The reported energy consumption corresponds to the dynamic (active) energy during computation. Although not considered here, the MCA's inherent nonvolatility—resulting in zero standby leakage—could offer an even greater energy efficiency advantage in practical deployments.
- **Throughput Estimation:** The maximum path delay extracted from synthesis results was used to determine the operating throughput of the benchmark CMOS hardware.

Supplementary Note 15

In large arrays, crosstalk may occur when neighboring MCA devices are placed too close together. The primary source of crosstalk is the dipolar (stray) field generated by the magnetic layer of adjacent devices.

To quantify this effect, we calculated the stray-field distribution based on the actual device dimensions: the magnetic layer (Domain Channel) measures 200 µm × 20 µm × 1 nm, with a saturation magnetization Ms=1000 emu/cc. The results are summarized in Supplementary Fig. 26. Supplementary Fig. 26a shows the device geometry, and Supplementary Fig. 26b maps the out-of-plane field component (Bz) near the device. The detailed dependence of stray-field magnitude on distance from the device edge is presented in Supplementary Figs. 26c and 26d (short and long edges, respectively), with zoomed-in views from 100 nm to 1000 nm shown in Supplementary Figs. 26e and 26f.

The stray field magnitudes at characteristic lengths are summarized below:

- **At 10 nm from the device edge:**

|Bz| ≈ **19–20 mT**; the tangential in-plane component is ≈ **3 mT** (that’s |By| at a long edge or |Bx| at a short edge)

- **At 100 nm:**

|Bz| ≈ **2.0 mT**; tangential in-plane ≈ **0.03 mT** (**30 µT**).

- **At 1 µm:**

|Bz| ≈ **0.19 mT**; tangential in-plane ≈ **0.0003 mT** (**0.3 µT**).

For comparison, the coercivity of our magnetic layer is ~5 mT. Therefore, a 1 μm lateral spacing ensures that the maximum stray field (~0.19 mT) remains well below the coercivity, effectively suppressing magnetic crosstalk between adjacent devices.

To further increase device density, 100 nm or smaller spacing can be adopted if the perpendicular magnetic anisotropy (PMA) is enhanced. This can be achieved, for example, by reducing the CoFeB thickness or by switching to material systems such as Pt/CoFeB/MgO or Pt/Co/AlOx, which exhibit coercivities exceeding 100 mT. Under such conditions, stray-field interactions would be negligible.

We note that this estimation represents the worst-case crosstalk scenario, assuming a uniformly magnetized single-domain state. In practical devices, however, the magnetization alternates across domains, producing flux closure and substantial field cancellation. Consequently, the actual magnetic crosstalk in large arrays is expected to be significantly weaker than this upper bound.

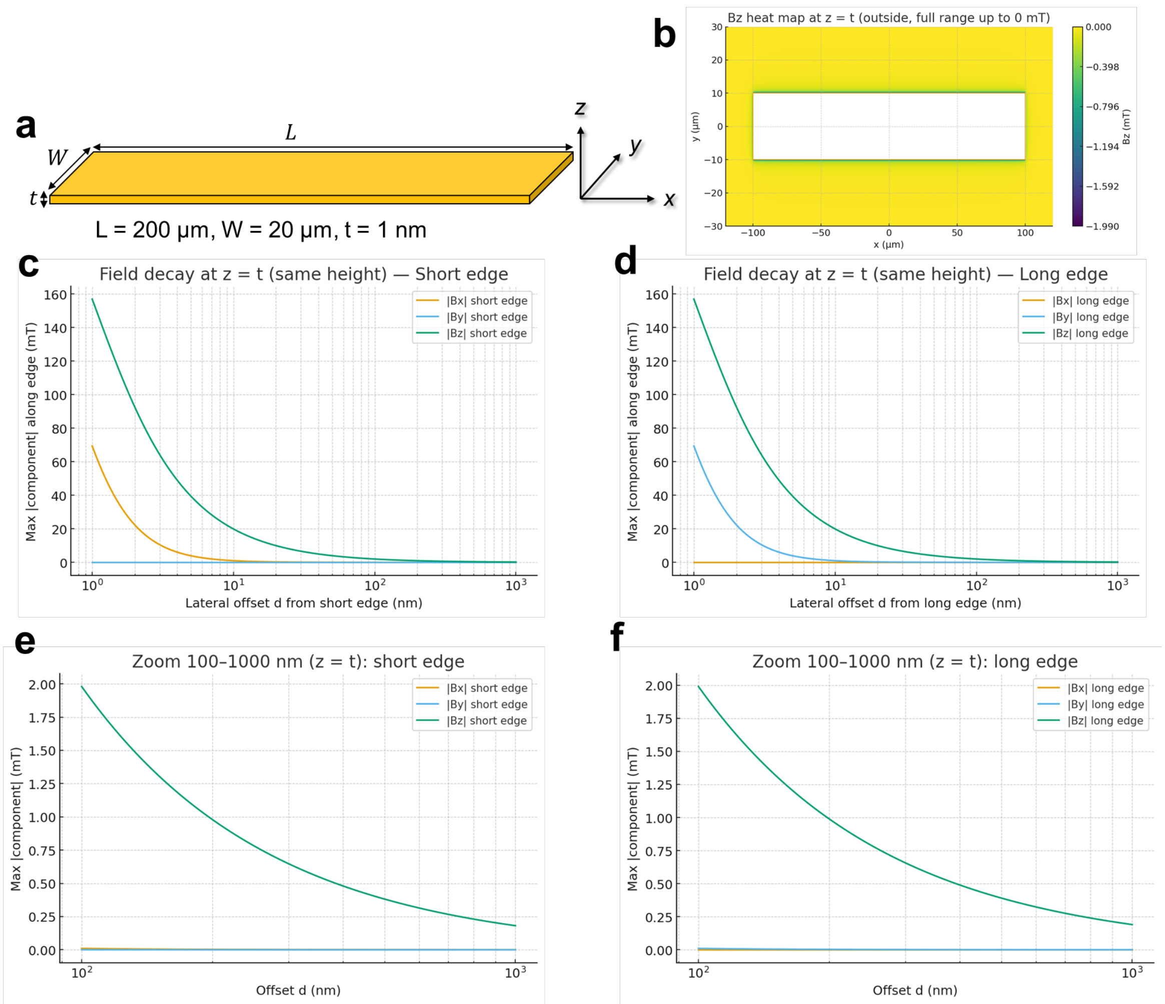

Supplementary Figure 26 | Stray field from the magnetic layer. (a), Device dimensions. **(b),** Stray field color map. **(c),** Stray field distribution from the short edge. **(d),** Stray field distribution from the long edge. **(e),** Zoom-in of (c) from 100nm to 1000nm. **(f),** Zoom-in of (d) from 100nm to 1000nm.

References

[1] Sala, G., Krizakova, V., Grimaldi, E. et al. Real-time Hall-effect detection of current-induced magnetization dynamics in ferrimagnets. Nat Commun 12, 656 (2021).

[2] Moretti, S., Voto, M. and Martinez, E., 2017. Dynamical depinning of chiral domain walls. *Physical Review B*, *96*(5), p.054433.

[3] Herranen, T. and Laurson, L., 2019. Barkhausen noise from precessional domain wall motion. *Physical review letters*, *122*(11), p.117205.

[4] Metaxas, P.J., Jamet, J.P., Mougin, A., Cormier, M., Ferré, J., Baltz, V., Rodmacq, B., Dieny, B. and Stamps, R.L., 2007. Creep and Flow Regimes of Magnetic Domain-Wall Motion in Ultrathin Pt/Co/Pt Films with Perpendicular Anisotropy. *Physical review letters*, *99*(21), p.217208.

[5] Chappert, C., Fert, A. & Van Dau, F. The emergence of spin electronics in data storage. Nature Mater 6, 813–823 (2007).

[6] Everspin Technologies Whitepaper. Fast Read/Write • Non-Volatile • Infinite Endurance. (Everspin Technologies, 2016).

[7] Kent, A., Worledge, D. A new spin on magnetic memories. Nature Nanotech 10, 187–191 (2015).

[8] Kan, J.J., Park, C., Ching, C., Ahn, J., Xue, L., Wang, R., Kontos, A., Liang, S., Bangar, M., Chen, H. and Hassan, S., 2016, December. Systematic validation of 2x nm diameter perpendicular MTJ arrays and MgO barrier for sub-10 nm embedded STT-MRAM with

practically unlimited endurance. In 2016 IEEE International Electron Devices Meeting (IEDM) (pp. 27-4). IEEE.

[9] Kan, J.J., Park, C., Ching, C., Ahn, J., Xie, Y., Pakala, M. and Kang, S.H., 2017. A study on practically unlimited endurance of STT-MRAM. IEEE Transactions on Electron Devices, 64(9), pp.3639-3646.

[10] Shridhar, K., Laumann, F. and Liwicki, M., 2019. A comprehensive guide to bayesian convolutional neural network with variational inference. *arXiv:1901.02731*.
